# Supplementary material for: Policies, practices, opportunities and challenges for tuberculosis screening: a global survey of national tuberculosis programmes
Source: BMJ Glob Health. 2025 Jul 22;10(7):e016000. doi: 10.1136/bmjgh-2024-016000 (PMC12281326; doi:10.1136/bmjgh-2024-016000)
Supplement: online supplemental file 1 [file bmjgh-10-7-s001.pdf]

Policies, practices, opportunities, and challenges for TB screening – A global survey of National TB Programmes.

Authors: Macpherson L, Miller C, Hamada Y, Rangaka MX, Ruhwald M, Falzon D, Kik S.V., Esmail H.

Supplementary Materials:

Table of Contents

Supplementary Table 1: List of participating countries and contributors. 2

Supplementary Table 2: List of eligible countries (reporting >1000 cases per year (2019) invited to participate including those who responded. 4

Supplementary Figure 1: Priority groups for screening for Tuberculosis (TB) disease for high and low TB burden countries. 8

Supplementary Figure 2: Representation of the tools reportedly used for Tuberculosis disease screening. 9

Supplementary Figure 3: Screening algorithms used by countries to screen for Tuberculosis disease. 10

Legend. 11

Copy of the initial email sent to eligible National TB Programmes 12

Survey PDF (in English). 14

Supplementary Table 1: List of participating countries and contributors.

Country	Role
**Angola**	Coordinator
**Botswana**	Public health specialist
**Equatorial Guinea**	Director
**Eritrea**	Manager
**Ethiopia**	Manager
**Gabon**	Public health specialist
**Gambia**	Manager
**Guinea**	Coordinator
**Kenya**	Officer
**Lesotho**	Manager
**Liberia**	Manager
**Malawi**	Manager
**Mozambique**	Other
**Niger**	Coordinator
**Nigeria**	Director
**Rwanda**	Director
**Somalia**	Manager
**Sierra Leone**	Manager
**South Africa**	Director
**Togo**	Director
**Uganda**	Manager
**United Republic of Tanzania**	Manager
**Zambia**	Manager
**Zimbabwe**	Director
**Brazil**	Coordinator
**Colombia**	Coordinator
**Ecuador**	Director
**Haiti**	Director
**Paraguay**	Director
**United States of America**	Director
**Venezuela (Bolivarian Republic of)**	Public health specialist
**Yemen**	Manager
**Iraq**	Director
**Morocco**	Manager
**Pakistan**	Coordinator
**Belgium**	Director
**France**	Director
**Georgia**	Director

**Germany**	Public health specialist
**Kazakhstan**	Director
**Lithuania**	Director
**Portugal**	Director
**Republic of Moldova**	Coordinator
**Romania**	Manager
**Serbia**	Coordinator
**Spain**	Coordinator
**Turkey**	Director
**United Kingdom**	Director
**Bangladesh**	Manager
**India**	Director
**Indonesia**	Manager
**Sri Lanka**	Medic
**Australia**	Director
**China**	Manager
**Japan**	Director
**Lao PDR**	Director
**Malaysia**	Director
**Philippines**	Other
**Singapore**	Director
**Viet Nam**	Director

Supplementary Table 2: List of eligible countries (reporting >1000 cases per year (2019) invited to participate including those who responded.

Red text indicates WHO high TB-burden country.
Regions: AFR: Africa, AMR: Americas, EMR: Eastern Mediterranean, EUR: European, SEAR: South-East Asia, WPR: Western Pacific.

Country	WHO Region	Proportion of global TB Burden (%)	Participation
Algeria	AFR	0.26	No
Angola	AFR	1.13	Yes
Benin	AFR	0.06	No
Botswana	AFR	0.06	Yes
Burkina Faso	AFR	0.10	No
Burundi	AFR	0.12	No
Cameroon	AFR	0.46	No
Central African Republic	AFR	0.26	No
Chad	AFR	0.23	No
Congo	AFR	0.20	No
Côte d'Ivoire	AFR	0.35	No
Democratic Republic of the Congo	AFR	2.80	No
Equatorial Guinea	AFR	0.03	Yes
Eritrea	AFR	0.03	Yes
Eswatini	AFR	0.04	No
Ethiopia	AFR	1.58	Yes
Gabon	AFR	0.11	Yes
Gambia	AFR	0.04	Yes
Ghana	AFR	0.44	No
Guinea	AFR	0.22	Yes
Guinea-Bissau	AFR	0.07	No
Kenya	AFR	1.41	Yes
Lesotho	AFR	0.14	Yes
Liberia	AFR	0.15	Yes
Madagascar	AFR	0.63	No
Malawi	AFR	0.27	Yes
Mali	AFR	0.10	No
Mauritania	AFR	0.04	No
Mozambique	AFR	1.11	Yes
Namibia	AFR	0.12	No
Niger	AFR	0.20	Yes

**Nigeria**	AFR	4.42	Yes
**Rwanda**	AFR	0.07	Yes
**Senegal**	AFR	0.19	No
**Sierra Leone**	AFR	0.23	Yes
**South Africa**	AFR	3.62	Yes
**South Sudan**	AFR	0.25	No
**Togo**	AFR	0.03	Yes
**Uganda**	AFR	0.88	Yes
**United Republic of Tanzania**	AFR	1.38	Yes
**Zambia**	AFR	0.59	Yes
**Zimbabwe**	AFR	0.29	Yes
**Argentina**	AMR	0.13	No
**Bolivia (Plurinational State of)**	AMR	0.12	No
**Brazil**	AMR	0.97	Yes
**Canada**	AMR	0.02	No
**Chile**	AMR	0.03	No
**Colombia**	AMR	0.18	Yes
**Dominican Republic**	AMR	0.05	No
**Ecuador**	AMR	0.08	Yes
**El Salvador**	AMR	0.04	No
**Guatemala**	AMR	0.05	No
**Haiti**	AMR	0.19	Yes
**Honduras**	AMR	0.03	No
**Mexico**	AMR	0.30	No
**Nicaragua**	AMR	0.03	No
**Panama**	AMR	0.02	No
**Paraguay**	AMR	0.03	Yes
**Peru**	AMR	0.39	No
**United States of America**	AMR	0.10	Yes
**Uruguay**	AMR	0.01	No
**Venezuela (Bolivarian Republic of)**	AMR	0.13	Yes
**Afghanistan**	EMR	0.72	No
**Djibouti**	EMR	0.02	No
**Egypt**	EMR	0.12	No
**Iran (Islamic Republic of)**	EMR	0.11	No
**Iraq**	EMR	0.16	Yes
**Libya**	EMR	0.04	No
**Morocco**	EMR	0.35	Yes
**Pakistan**	EMR	5.73	Yes
**Saudi Arabia**	EMR	0.03	No
**Somalia**	EMR	0.40	Yes
**Sudan**	EMR	0.29	No

**Syrian Arab Republic**	EMR	0.03	No
**Tunisia**	EMR	0.04	No
**Yemen**	EMR	0.14	Yes
**Azerbaijan**	EUR	0.06	No
**Belarus**	EUR	0.03	No
**Belgium**	EUR	0.01	Yes
**Bulgaria**	EUR	0.01	No
**France**	EUR	0.06	Yes
**Georgia**	EUR	0.03	Yes
**Germany**	EUR	0.05	Yes
**Italy**	EUR	0.04	No
**Kazakhstan**	EUR	0.13	Yes
**Kyrgyzstan**	EUR	0.07	No
**Lithuania**	EUR	0.01	Yes
**Poland**	EUR	0.06	No
**Portugal**	EUR	0.02	Yes
**Republic of Moldova**	EUR	0.03	Yes
**Romania**	EUR	0.13	Yes
**Russian Federation**	EUR	0.73	No
**Serbia**	EUR	0.01	Yes
**Spain**	EUR	0.04	Yes
**Tajikistan**	EUR	0.08	No
**Turkey**	EUR	0.13	Yes
**Turkmenistan**	EUR	0.03	No
**Ukraine**	EUR	0.34	No
**United Kingdom**	EUR	0.05	Yes
**Uzbekistan**	EUR	0.22	No
**Bangladesh**	SEAR	3.63	Yes
**Bhutan**	SEAR	0.01	No
**China, Hong Kong SAR**	SEAR	0.05	No
**Democratic People's Republic of Korea**	SEAR	1.33	No
**India**	SEAR	26.55	Yes
**Indonesia**	SEAR	8.50	Yes
**Myanmar**	SEAR	1.75	No
**Nepal**	SEAR	0.68	No
**Sri Lanka**	SEAR	0.14	Yes
**Thailand**	SEAR	1.06	No
**Timor-Leste**	SEAR	0.06	No
**Australia**	WPR	0.02	Yes
**Cambodia**	WPR	0.47	No
**China**	WPR	8.38	Yes

**Japan**	WPR	0.17	Yes
**Lao People's Democratic Republic**	WPR	0.11	Yes
**Malaysia**	WPR	0.29	Yes
**Mongolia**	WPR	0.14	No
**Papua New Guinea**	WPR	0.38	No
**Philippines**	WPR	6.02	Yes
**Republic of Korea**	WPR	0.30	No
**Singapore**	WPR	0.02	Yes
**Viet Nam**	WPR	1.71	Yes

Supplementary Figure 1: Priority groups for screening for Tuberculosis (TB) disease for high and low TB burden countries.

Priority Groups for Screening for Active TB: High TB burden countries (n=21).

Population Groups	High priority	Neutral	Low priority	Not applicable
Household contacts (children)	95%	0%	5%	0%
People living with HIV	95%	5%	0%	0%
Household contacts (adults)	90%	0%	10%	0%
Prisons and penitentiary institutions	86%	14%	0%	0%
Urban poor communities	71%	19%	10%	0%
People with clinical risk factors other than HIV seen in healthcare	67%	29%	5%	0%
Other vulnerable or marginalized groups with limited access to healthcare	67%	10%	19%	0%
General population with high TB prevalence (>0.5%)	62%	10%	10%	14%
Communities in remote or isolated areas	62%	10%	24%	5%
Current and former workers in work places with silica exposure	52%	29%	5%	10%
Refugees	48%	10%	19%	19%
Migrants	38%	19%	19%	14%
Homeless communities	38%	29%	14%	10%
Internally displaced people	38%	14%	19%	24%
People with untreated fibrotic lesions on CXR	33%	48%	14%	0%
Indigenous populations	29%	14%	24%	29%

Priority Groups for Screening for Active TB: Lower TB burden countries (n=39).

Population Groups	High priority	Neutral	Low priority	Not applicable
Household contacts (children)	90%	8%	3%	0%
People living with HIV	77%	10%	10%	3%
Household contacts (adults)	85%	10%	3%	0%
Prisons and penitentiary institutions	87%	5%	5%	3%
Urban poor communities	28%	36%	23%	13%
People with clinical risk factors other than HIV seen in healthcare	62%	15%	13%	10%
Other vulnerable or marginalized groups with limited access to healthcare	41%	28%	15%	15%
General population with high TB prevalence (>0.5%)	38%	18%	10%	26%
Communities in remote or isolated areas	26%	21%	23%	31%
Current and former workers in work places with silica exposure	33%	23%	15%	28%
Refugees	46%	23%	8%	23%
Migrants	54%	21%	10%	15%
Homeless communities	36%	26%	10%	28%
Internally displaced people	23%	28%	15%	33%
People with untreated fibrotic lesions on CXR	33%	38%	18%	10%
Indigenous populations	23%	21%	5%	49%

Supplementary Figure 2: Representation of the tools reportedly used for Tuberculosis disease screening.

**Symptom(s) used for screening**	**Population:*** Adult contacts	Child contacts	PLHIV
TB symptoms used for screening	46/56 (82%)<br>18/19 (95%)	45/56 (80%)<br>18/19 (95%)	45/56 (80%)<br>18/19 (95%)
Any cough	22/46 (48%)<br>8/18 (44%)	28/45 (62%)<br>10/18 (56%)	39/45 (87%)<br>15/18 (83%)
Prolonged cough	36/46 (78%)<br>14/18 (78%)	30/45 (67%)<br>9/18 (50%)	28/45 (62%)<br>8/18 (44%)
Any TB symptoms	39/46 (85%)<br>14/18 (78%)	41/45 (91%)<br>16/18 (89%)	42/45 (93%)<br>15/18 (83%)
**CXR use**			
CXR used for screening	43/56 (77%)<br>15/19 (79%)	42/56 (75%)<br>15/19 (79%)	41/56 (73%)<br>15/19 (79%)
Following +ve symptom screen	12/43 (28%)<br>2/15 (13%)	7/42 (16%)<br>2/15 (13%)	8/41 (20%)<br>2/15 (13%)
For all / following negative symptom screen	31/43 (72%)<br>13/15 (87%)	35/42 (83%)<br>13/15 (87%)	33/41 (80%)<br>13/15 (87%)

All countries (n=56)
High TB-burden n(=19)

* 4 countries have been excluded: 3 who did not perform systematic screening and 1 that did not provide answers for this section therefore, data for 56 countries including 19 high burden countries are reported here.

Supplementary Figure 3: Screening algorithms used by countries to screen for Tuberculosis disease.

Number of countries using screening tools (total n=50*)

Adult contacts	**8**	**11**	**3**	**24**	**4**
Child contacts	**9**	**7**	**1**	**29**	**3**
PLHIV	**9**	**6**	**3**	**28**	**4**

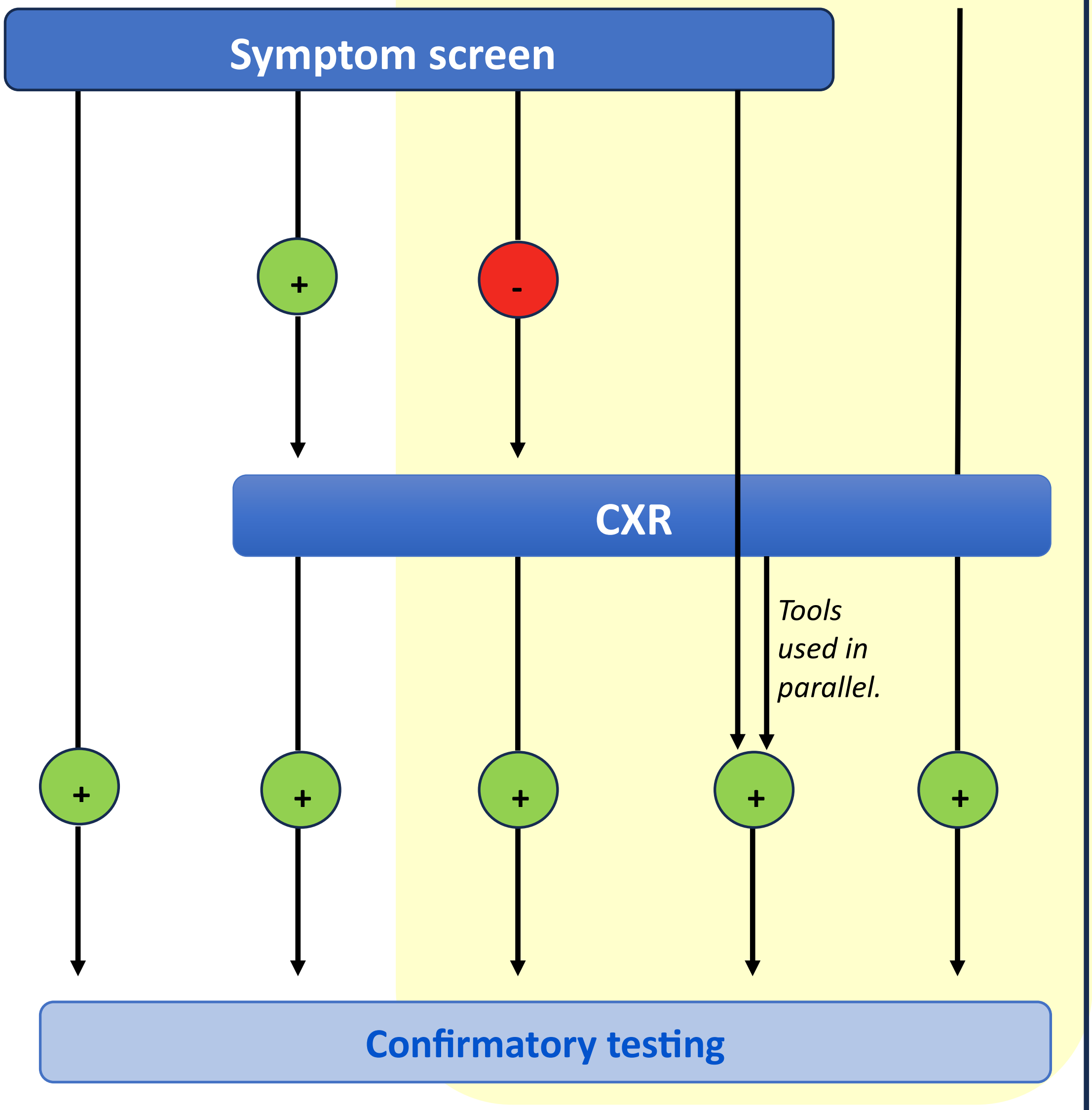

Supplementary figure 4 shoes how countries reported their use of different screening tools for three of the key risk groups – adult TB contacts, child TB contacts and people living with HIV (PLHIV). The yellow box highlights 3 algorithms that would detect subclinical Tuberculosis.

* Exclusions from this figure: 3 countries reported that they did not perform TB screening, 1 did not complete this section of the survey, 1 country provided discrepant answers to these questions, and 5 reported no use of either symptoms or CXR for TB screening.

Legend.

Supplementary Table 1: List of participating countries and contributors.
Showing a list of participating countries and acknowledging their contributors.

Supplementary Table 2: List of eligible countries invited to participate including those who responded.
Showing a list of eligible countries (who reported >1000 Tuberculosis cases in 2019) who were invited to participate in the survey. The column on the left indicated whether the country actually participated in the survey.

Supplementary Figure 1: Priority groups for screening for Tuberculosis disease.
Showing the priority groups for screening for Tuberculosis disease for both high and lower burden countries.

Supplementary Figure 2: Representation of tools used for Tuberculosis disease screening.
This is a visual representation of the types of screening tools used by participating countries to screen for Tuberculosis disease.

Supplementary Figure 3: Screening algorithms used by countries to screen for Tuberculosis disease.
This is a visual representation of screening algorithms used by countries to screen for Tuberculosis disease.

Copy of the initial email sent to eligible National TB Programmes

Dear National TB Programme colleagues,

We are contacting you to invite you to take part in global survey on behalf of your country's National TB Programme. The aim of the survey is to understand the policies, practices, and challenges of **screening for active TB**. The survey is being sent to all NTPs in countries that report >1000 TB cases per year.

This survey has been developed by a team from University College London, FIND and the World Health Organisation following the recently updated WHO guidelines on systematic screening. The survey is researcher-led and has received ethical approval from the University College London Research Ethics Committee.

The link to the survey which has 23 main questions is below. We estimate it will take approximately 20-25 minutes to complete if the information is to hand.

LINK TO THE SURVEY: **Policies, practices and challenges of systematic screening for active TB: A global survey**

We only require one response from each NTP and it can be completed by anyone with knowledge of their country's policies and practices around systematic screening for TB. We have attached a PDF of the survey so the questions can be viewed in advance if preferred, but the survey should be completed online. We would like as many of the invited NTPs as possible to complete this survey, so that it is truly representative, and ask you to do so by **Monday 13th December 2021.**

Why are we conducting this survey now?
Systematic screening for active TB, to detect and treat TB early, is a central component of the WHO End TB strategy. It is particularly relevant now in the context of mitigating and reversing the impact COVID has had on TB case notification. The WHO updated their guidance for systematic screening in 2021 providing 17 new and revised recommendations and an operational handbook to help implementation. This guidance forms the basis for the survey.

We recognise that countries are at different stages of implementing their screening programmes for active TB and that each country faces challenges, some of which will be common. This survey will provide information on the current screening practices and policies in different countries and identify the common challenges. This will be useful to policymakers, funding agencies and academics involved in maximising effectiveness of screening for active TB.

What will we do with the results?
The results of the survey will be shared with those who participate, so you can contextualise your experiences regionally and globally. In addition, a report will be provided to the regional WHO offices and WHO Geneva. The work will subsequently be submitted for publication in which we will acknowledge all contributors. We will not publish individual country data but will collate responses. If you have specific concerns about data sharing, please contact us.

Many thanks for your participation.
For any queries please contact: mrcctu.ntpsurvey@ucl.ac.uk.

Yours sincerely,

Dr Hanif Esmail
On behalf of the study team.

Study collaborators:
Sandra Kik (FIND), Morten Ruhwald (FIND), Cecily Miller (WHO), Dennis Falzon (WHO)

THIS PDF IS FOR REFERENCE ONLY. Please complete the survey online.

Policies, practices, and challenges of systematic screening for TB disease: a global survey.

Introduction

Thank you for taking part in this global survey on behalf of your country's National TB Programme. The aim is to understand the policies, practices, and challenges of systematic screening for active TB disease in countries that report >1000 TB cases per year. This will provide important information for policymakers, funding agencies and academics involved in maximising the effectiveness of screening for active TB disease.

The survey has been developed by a team from University College London, FIND and WHO and has received ethical approval from the University College London Research Ethics Committee. The 2021 WHO guideline on systematic screening for TB disease serves as the benchmark for many of the questions.

The questions focus on systematic screening for active TB disease in your country (as opposed to screening for latent TB infection (LTBI)). We estimate that it will take you approximately 20 minutes to complete.

<u>For the purposes of this survey systematic screening refers to:</u> **The systematic identification of active TB disease in people who are not seeking healthcare, through assessing symptoms and using tests, examinations or other procedures that can be applied rapidly. For those who screen positive, the diagnosis of TB disease then needs to be established by one or several diagnostic tests and additional clinical assessments. This is also known as active case finding and is distinguished from testing for latent TB infection.**

For any queries please contact: mrcctu.NTPsurvey@ucl.ac.uk

THIS PDF IS FOR REFERENCE ONLY. Please complete the survey online.

Policies, practices, and challenges of systematic screening for TB disease: a global survey.

General information

1. Which country's National TB Programme (NTP) are you answering on behalf of?

2. Your job title and affiliation(s)

3. Please state your name and contact details. *This information will only be used to contact you about your survey responses and will not be shared with any third parties.*

Name

Email Address

Phone Number

THIS PDF IS FOR REFERENCE ONLY. Please complete the survey online.

Policies, practices, and challenges of systematic screening for TB disease: a global survey.

Policies and practices in relation to systematic screening for TB disease in your country

The questions in this section focus on your country's policies and practices in relation to systematic screening for active TB disease (not LTBI).

4. Prior to this survey, were you aware of the 2021 WHO Guidelines on systematic screening for active TB disease?

- ◯ Yes
- ◯ No

5. Is systematic screening for active TB disease conducted in any form in your country?

- ◯ Yes
- ◯ No

6. Do you have a national strategic plan for TB

- ◯ Yes
- ◯ No

If answered "Yes" please state what time period the strategic plan covers

[ ]

7. Does your national strategic plan for TB include a plan to either increase or decrease systematic screening for active TB disease?

- ◯ Not applicable (no national strategic plan)
- ◯ Increase systematic screening
- ◯ Decrease systematic screening
- ◯ No plan to increase or decrease
- ◯ Don't know
- ◯ Other (please specify)

[ ]

8. Do you have an existing national guideline that covers systematic screening for active TB disease?

- ◯ Yes
- ◯ No
- ◯ No - but we have a national policy or guideline in progress
- ◯ Other (please specify)

9. In relation to implementing systematic screening for active TB disease which of the following steps have been carried out or are currently being carried out? *Please answer regardless of whether you have an existing policy for screening. - see chapter 2 of the WHO operational handbook for more information.* [Tick all that apply]

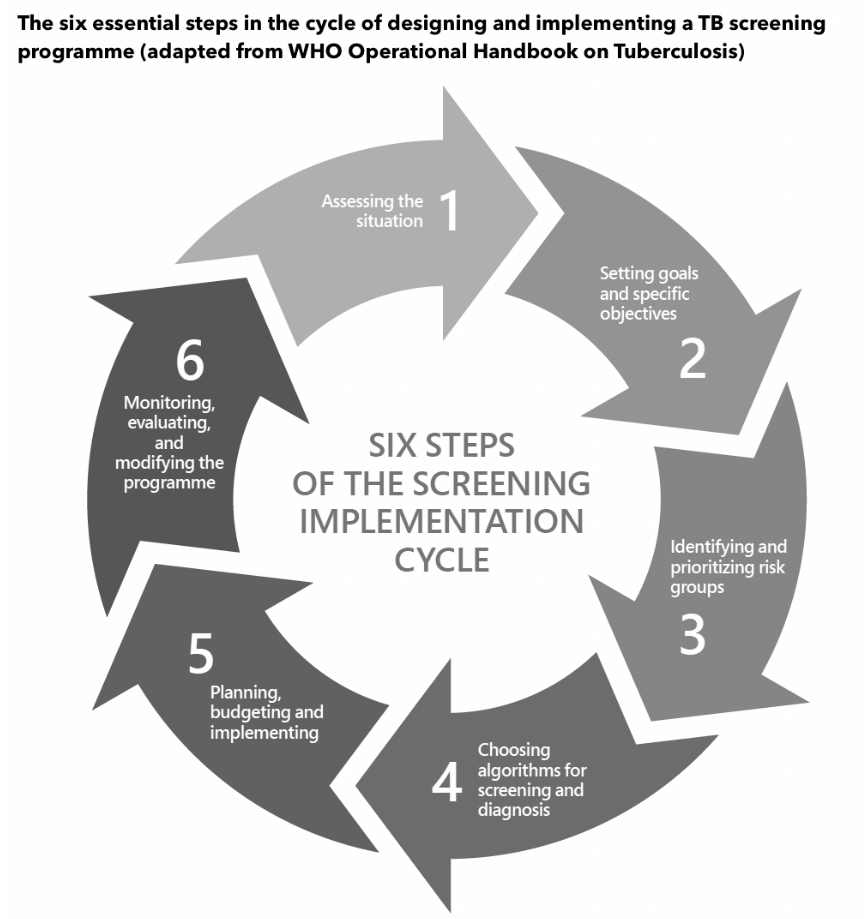

- ☐ Situation assessment
- ☐ Setting goals and specific objectives
- ☐ Identifying and prioritizing risk groups
- ☐ Choosing algorithms for screening and diagnosis
- ☐ Budgeting
- ☐ Monitoring and evaluation
- ☐ None of the above
- ☐ Not applicable

10. Please rate the priority of the following groups for systematic screening for active TB disease in your country? *Please answer regardless of whether you have an existing policy for screening.*

	Low priority	Neutral	High priority	Not applicable
General population with high TB prevalence (>0.5%)	○	○	○	○
People living with HIV	○	○	○	○
Household contacts (adults)	○	○	○	○
Household contacts (children)	○	○	○	○
Prisons and penitentiary institutions	○	○	○	○
Current and former workers in work places with silica exposure	○	○	○	○
People with clinical risk factors other than HIV seen in healthcare	○	○	○	○
People with untreated fibrotic lesions on CXR	○	○	○	○
Urban poor communities	○	○	○	○
Homeless communities	○	○	○	○
Communities in remote or isolated areas	○	○	○	○
Indigenous populations	○	○	○	○
Migrants	○	○	○	○
Refugees	○	○	○	○
Internally displaced people	○	○	○	○
Other vulnerable or marginalized groups with limited access to healthcare	○	○	○	○

11. In which of the following groups do you currently have a policy for systematic screening for active TB disease? [Tick all that apply]

- [ ] Not applicable (no policy for systematic screening in place)
- [ ] General population with high TB prevalence (e.g. >0.5%)
- [ ] People living with HIV
- [ ] Household contacts (adults)
- [ ] Household contacts (children)
- [ ] Prisons and penitentiary institutions
- [ ] Current and former workers in work places with silica exposure
- [ ] People with clinical risk factors other than HIV seen in healthcare
- [ ] People with untreated fibrotic lesions on CXR
- [ ] Other (please specify)
- [ ] Urban poor communities
- [ ] Homeless communities
- [ ] Communities in remote or isolated areas
- [ ] Indigenous populations
- [ ] Migrants
- [ ] Refugees
- [ ] Internally displaced people
- [ ] Other vulnerable or marginalized groups with limited access to healthcare

12. Which stakeholders or organisations in your country are the main providers of systematic screening for active TB? [Tick all that apply]

- [ ] Government/ public health
- [ ] Private sector
- [ ] Non-Governmental Organisation
- [ ] Academic institution
- [ ] Other (please specify)

13. Approximately what proportion of screening activity for active TB disease, regardless of who provides it, is reported to the National TB programme?

- ◯ Not applicable (no screening activity)
- ◯ All (100%)
- ◯ Majority (75%)
- ◯ Half (50%)
- ◯ Minority (25%)
- ◯ None (0%)

If all screening activity is not reported to NTP please comment on what the challenges are

[ ]

14. What data does your NTP routinely collect for monitoring and evaluating your systematic screening programme for active TB disease? [Tick all that apply]

- ☐ No data is collected for monitoring of systematic screening
- ☐ Number of people eligible for screening according to national guidance
- ☐ Number of people screened for TB (total)
- ☐ Number of people screened by risk group (e.g. contacts, PLHIV, migrants etc)
- ☐ Number of people screened by the screening tool used (e.g. CXR vs symptoms only)
- ☐ Number of people who screen positive for TB (i.e. requiring confirmatory testing)
- ☐ Number of people evaluated for TB disease (i.e. receiving confirmatory testing)
- ☐ Number of people diagnosed with TB (total, including clinical and bacteriological diagnosis)
- ☐ Number of people diagnosed with TB by bacteriological status (i.e. confirmed or not)
- ☐ Number of people diagnosed with TB classified by symptom status
- ☐ Number of people started on TB treatment
- ☐ Number of people successfully completing TB treatment
- ☐ Other (please specify)

[ ]

15. Is systematic screening for active TB disease budgeted in your National TB budget?

- ◯ Not applicable (no screening)
- ◯ Yes
- ◯ No
- ◯ Partially
- ◯ Don't know
- ◯ Other (please specify)

16. What is the source of funding within the National TB budget for systematic screening activity for active TB?

- ◯ Not applicable
- ◯ Domestic funding
- ◯ International donor funding (including the Global Fund)
- ◯ Mixture of domestic and international funding
- ◯ Unfunded
- ◯ Other (please specify)

Please specify other sources of funding and describe the approximate proportion of funding if mixed, for example: 50% domestic funding, 50% international.

17. Going forward, has the priority for systematic screening for active TB changed due to the COVID pandemic?

- ◯ Increased priority
- ◯ Not affected priority
- ◯ Reduced priority
- ◯ Don't know
- ◯ Other

Please explain your answer

THIS PDF IS FOR REFERENCE ONLY. Please complete the survey online.

Policies, practices, and challenges of systematic screening for TB disease: a global survey.

Tools and algorithms used for systematic screening for active TB disease in your country

The questions in this section focus on your country's use of screening tools for active TB disease, focusing on the following population groups: Adult contacts; child contacts; people living with HIV (PLHIV); and other relevant risk groups you feel are screened differently for active TB in your country.

For the purposes of this survey the following definitions apply:
<u>Prolonged cough:</u> Cough lasting ≥ 2 weeks (please use the comments box to detail any alternative definitions e.g. if your country defines prolonged cough as lasting ≥ 3 weeks).
<u>Any TB symptoms:</u> Cough of any duration, sputum, haemoptysis, fever, night sweats or weight loss.
<u>W4SS:</u> WHO-recommended four-symptom-screen, comprising screening for a current cough, fever, night sweats or weight loss.

18. Which symptoms do you use to screen for active TB disease? This applies to the following populations: Adult contacts of TB cases, child contacts of TB cases, PLHIV, and any other relevant risk groups. [Please select 'yes' from the dropdown menu where applicable, otherwise leave the box blank]

	Adult contacts	Child contacts	PLHIV	Other risk groups
Prolonged cough				
Any cough				
Any TB symptom				
W4SS (for PLHIV only)				
We do not ask about symptoms				

Comments (if applicable). For example, if you use symptoms to screen in any other population group or scenario, please provide that information here.

19. Where do you use CXR in your screening algorithm for active TB disease? This applies to the following populations: Adult contacts of TB cases, child contacts of TB cases, PLHIV, and other relevant risk groups. [Please select 'yes' from the dropdown menu where applicable, otherwise leave the box blank]

	Adult contacts	PLHIV	Children contacts	Other risk groups
All people being screened				
People with a positive symptom screen (see question 17)				
People with a negative symptom screen (see question 17)				
People with a positive bacteriological test				
We do not use CXR as a screening tool in this context				

Comments (if applicable). For example, if you use CXR to screen in any other population group or scenario, please provide that information here.

20. Do you use molecular diagnostics (Xpert MTB/RIF, Xpert Ultra or Truenat) as **screening tests** for active TB disease (as opposed to as confirmatory tests)? This applies to the following populations: Contacts of TB cases, PLHIV and any 'other' groups. [Please select 'yes' from the dropdown menu where applicable, otherwise leave the box blank]

	Contacts	PLHIV	Other
All people being screened			
All medical inpatients where TB prevalence is high (for PLHIV only)			
We do not use molecular diagnostics as screening tests for active TB			

Comments (if applicable). For example, if you use molecular diagnostics to screen in any other population group or scenario, please provide that information here.

21. Do you use CRP to screen for active TB disease for PLHIV in your country?

- ◯ Yes
- ◯ No
- ◯ Don't know

Comments (if applicable)

22. What **confirmatory bacteriological tests** for active TB disease are routinely used in people screening positive in your country? [Tick all that apply]

- ☐ Not applicable
- ☐ Smear
- ☐ Culture (MGIT)
- ☐ Culture (solid)
- ☐ Molecular (Xpert MTB/RIF)
- ☐ Molecular (Xpert Ultra)
- ☐ Molecular (Truenat)
- ☐ Molecular (other - please specify in the comments box below)
- ☐ Not stated in guidelines
- ☐ Other (please specify in the comments box below)

Comments (if applicable)

THIS PDF IS FOR REFERENCE ONLY. Please complete the survey online.

Policies, practices, and challenges of systematic screening for TB disease: a global survey.

The use of CXR technologies for screening for active TB disease in your country

The questions in this section focus on the use of CXR technologies in your country.

For the purposes of this survey the following definitions apply:

<u>Computer-Aided Detection (CAD):</u> The use of specialised software to interpret abnormalities on digital chest radiographs that are suggestive of TB. The results are expressed as abnormality scores.

23. Is implementing or expanding the use of CXR for systematic screening for active TB disease part of your national strategic plan?

- ○ Yes
- ○ No
- ○ Don't know

24. What are the barriers to implementing or expanding CXR based systematic screening for active TB disease in your country? *Please answer regardless of whether you have an existing policy for screening.* [Tick all that apply]

- ☐ High equipment costs
- ☐ Funding
- ☐ Access to radiologists
- ☐ Access to radiographers
- ☐ Lack of laboratory infrastructure for bacteriological testing following CXR
- ☐ Regulatory or procurement issues
- ☐ Perceived limited impact of CXR screening
- ☐ Concerns about increases in empirical TB treatment
- ☐ Patient factors (e.g cost to individual)
- ☐ Safety concerns regarding radiation dose
- ☐ Low community access to CXR
- ☐ There are no barriers
- ☐ Other (please specify)

25. If CXR is used in systematic screening for active TB, is CAD used to inform management decisions?

- ○ Not applicable - not using CXR for screening
- ○ No - using CXR from screening but not using CAD
- ○ No – but planning to pilot use of CAD
- ○ Only using in research settings or as a pilot
- ○ Yes - using in some private sector settings
- ○ Yes - using in some public sector settings
- ○ Yes - in most settings for TB screening

Comment (if applicable)

26. What are the barriers and concerns regarding implementation of CAD in screening for active TB in all settings? [Tick all that apply]

- ☐ We are not aware of this technology
- ☐ Digital CXR not currently being used
- ☐ Lack of validation in all age groups
- ☐ Sufficient expertise and availability of radiologists exists
- ☐ Too expensive
- ☐ Poor specificity
- ☐ Poor predictive value
- ☐ Regulatory issues
- ☐ Connectivity (internet) issues
- ☐ Procurement issues
- ☐ Insufficient infrastructure
- ☐ Concerns about data security
- ☐ There are no barriers
- ☐ Other (please specify)

Thank you for taking the time to complete this survey.

27. Are you happy for us to contact you about your answers?

- ◯ Yes
- ◯ No

Survey PDF (in English).
